# Long lasting response of trabectedin in patient with gastric leiomyosarcoma with liver metastasis: an update to previous report

**DOI:** 10.2144/fsoa-2019-0085

**Published:** 2019-12-09

**Authors:** Sameer Rastogi, Kaushal Kalra, Parisa Manasa, Monali Rajawat, Varshil Mehta

**Affiliations:** 1 Department of Medical Oncology, All India Institute of Medical Sciences, New Delhi 110029, India; 2 Sachin Sarcoma Society, New Delhi 110036, India; 3 Sheffield Teaching Hospitals NHS Foundation Trust, Northern General Hospital, Sheffield S5 7AU, UK; 4 Medkrux Research Group, Mumbai 400064, India; 5 Chelsea and Westminster Hospital NHS Foundation Trust, West Middlesex University Hospital, Isleworth TW7 6AF, UK

**Keywords:** gastric LMS, liposarcoma, LMS, metastasis, pazopanib, trabectedin

## Abstract

Leiomyosarcoma of the stomach is an extremely rare malignancy for which treatment in advanced disease is hardly reported. Here, we report a case of a 48-year-old man with metastatic gastric Leiomyosarcoma who had previously received a combination of gemcitabine and docetaxel followed by pazopanib after detection of metastasis. The patient was started on trabectedin as per protocol and had disease control continuing for 17 cycles of trabectedin. His quality of life and absence of significant toxicities highlight the noncumulative nature of trabectedin and potential benefit in responding cases.

## Lay abstract

Leiomyosarcoma (LMS) is a very rare form of tumor which, when it spreads to other organs, can be dreadful. Here, we report a case of a 48-year-old man who was diagnosed with LMS of the stomach that subsequently affected the liver. Multiple lines of anti-cancer drugs were given to the patient. At present, the patient takes trabectedin and has been tolerating it very well. Most drugs have cumulative toxicity when given for a long period of time but trabectedin does not possess this effect, which is why we feel that trabectedin should be considered as first-line treatment for LMS in the future.

In the last few years, the landscape of sarcoma management has changed with the approval of new and better agents. After pazopanib was approved in nonadipocytic sarcomas, trabectedin received US FDA approval in October, 2015 for the treatment of advanced L-sarcomas (liposarcoma and leiomyosarcoma [LMS]) in patients previously treated with anthracyclines. LMS is one of the most common soft tissue sarcomas and arises from smooth muscle cells. Both the approval trials of pazopanib and trabectedin had LMS as the most common subtype [1,2].

Patients treated with trabectedin had longer progression-free survival (PFS) than patients who received dacarbazine (4.2 vs 1.5 months; p = 0.001). However, there was no difference in overall survival (OS) in the two arms [2].

Trabectedin has a pleiotropic mechanism of action including the interaction with the minor groove of the DNA double helix, affecting transcription of different genes involved in DNA repair, facilitating lethal DNA strand breaks, directing growth inhibition and resulting in the death of malignant cells. It also displays anti-inflammatory and immunomodulatory properties because of the hinderance of factors that promote tumor growth, angiogenesis and metastasis [3,4]. Some of the most common adverse effects (all grades) of trabectedin in registration trials were nausea (73%), fatigue (67%), neutropenia (49%), increased AAT (39%), vomiting (44%), anemia (39%) etc [2].

Here, we report an unusual case of gastric LMS that had earlier responded to pazopanib [5] and as subsequent therapy had long-lasting response to trabectedin.

## Case presentation

A 48-year-old man having no co-morbidities was diagnosed with LMS of stomach (greater curvature) in his local area and underwent mass excision for the same in December, 2014. The histopathology was suggestive of high-grade LMS. The patient received adjuvant chemotherapy (6 cycles) of doxorubicin. After a treatment-free interval of 18 months, he developed metastasis to the liver in September, 2016. The patient further was given gemcitabine–docetaxel (7 cycles), after which the disease progressed. Subsequently, the patient came to our institute for further therapy. At presentation, he had Eastern Cooperative Oncology Group Performance Status (ECOG PS) 1 and we then started him on pazopanib 400 mg.

The patient had partial response initially on pazopanib (dosage: 400 mg OD) but after 6 months of therapy, the disease progressed. Hence, he was started on injection trabectedin from February 2018 onwards. Trabectedin was given at the dose of 1.5 mg/m2 on every 21^st^ day (every 3 weeks). Post three cycles of trabectedin, a partial response was observed. After eight cycles, a stable disease was noted in imaging by RECIST 1.1 criteria. He tolerated trabectedin well and had fatigue as the only toxicity (Grade 1), while adverse reactions such as alopecia and mucositis never developed during the treatment. The last cycle of trabectedin was given in May 2019 (17th cycle) and the imaging scans showed stable disease. His current ECOG PS is 1 and doing well on therapy.

**Figure 1.**
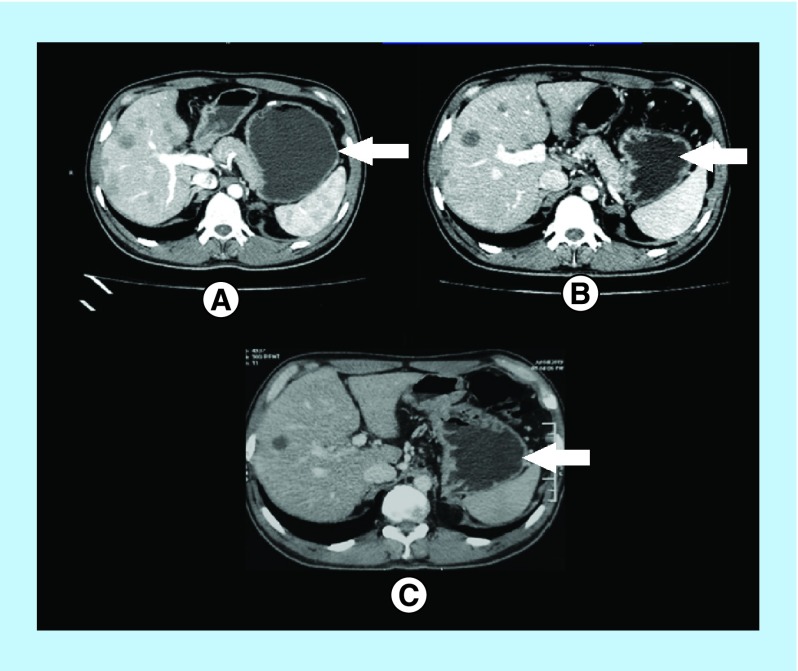
Computed tomography scan showing the largest lesion: (A) before start of the treatment; (B) post 3 cycles; (C) post 17 cycles.

The computed tomography scan before initiation of the treatment displayed the largest lesion with the dimension of 30.0 mm × 22.5 mm (Figure 1A), which subsequently reduced to 20 mm × 10 mm and 16 mm × 14 mm post 3 cycles (Figure 1B) and 17 cycles from the initiation of the therapy (Figure 1C) respectively. The disease is currently stable at 58 months after diagnosis of primary LMS was made.

## Discussion

We here describe a very rare case of a patient with primary gastric LMS with secondary metastasis to liver, who was treated with multiple lines of chemotherapy. The gastric LMS is an extremely rare malignant tumor, comprising only 1% of all the gastric malignancies. In an analysis of the Surveillance, Epidemiology and End Results (SEER) database in patients with primary gastric LMS diagnosed between 1988 to 2008, the median OS of gastric LMS was significantly higher than gastric adenocarcinoma (36 vs 10 months, p = 0.001) [6].

As mentioned in previous reports, the closest differential diagnosis of gastric LMS is gastrointestinal stromal tumor; however, the pathology and immunohistochemistry of our patient had been reviewed elaborately and was suggestive of LMS [5]. As this patient responded to a combination of gemcitabine–docetaxel, pazopanib and trabectedin, it is possible that the behavior of gastric LMS is similar to extremity LMS.

The patient has nonprogressive disease on 17th cycle of trabectedin with preserved quality of life and minimal toxicities, underlining the potential benefit of trabectedin in metastatic LMS. The minimal toxicity profile in our patient further reinforces the fact that the toxicity of trabectedin is non-cumulative as has been shown in previous trials [7–10].

In the trial by Demetri *et al.*, it was reported that 10% of patients were able to take 12 cycles of trabectdine continuously as compared with 2% of patients which took dacarbazine, thus a considerably smaller number of patients continues beyond 12 cycles [1]. Tavella *et al.* reported a case of uterine LMS, in which the patient was treated with 30 cycles of trabectedin and had a stable disease till the case was reported [11]. Similarly, in a study by Nteli *et al.*, the patient with uterine LMS had a stable disease till ninth cycle of chemotherapy [12]. In a report by Hauslbauer *et al.*, trabectedin provided 22 months of progression-free time with good quality of life in an elderly man with LMS with multiple comorbidities [13]. In a report by Corrado *et al.*, the best response to trabectedin happened after 9 cycles in a patient with metastatic inguinal LMS, signifying late response even after three to six cycles [14]. To our best knowledge there is no report of use of trabectedin in gastric LMS.

Since only a fraction of patients show clinical improvement after being exposed to trabectedin, it is imperative to use a biomarker in order to delineate the subgroup of the patients that will derive maximum benefit from the study. In a few clinical pharmacogenomic retrospective studies, it was shown that BRCA1 status could be predictive of trabectedin efficacy in sarcoma patients, which was later on refuted in the EORTC 62091 study in which prospective study of BRCA 1 assessment in soft tissue sarcoma was done (BRCA study) [15].

### Newer modalities of treatment options available for LMS

In the last few years various drugs like olaratumab, eribulin and some immunotherapies have also been tried in LMS. Olaratumab was approved in October 2016 in first-line setting in combination with doxorubicin, based upon a Phase Ib/II trial showing the dramatic OS benefit of 11.8 months as compared with single agent doxorubicin, which was unprecedented, leading to both reassurance and speculations after FDA accelerated approval [16]. This was followed by a Phase III placebo control randomized trial (ANNOUNCE), which used OS as primary end point. However, this trial failed to show OS benefit, with OS 20.4 months in the olaratumab/doxorubicin arm as compared with 29.8 months in the doxorubicin/placebo arm [17].

In a Phase III trial by Schoffski *et al.*, patients with advanced L-sarcomas who previously received two lines of chemotherapy were randomized to eribulin and dacarbazine [18]. The primary end point was OS while secondary end points were progression-free survival and progression-free rate. There was statistically significant improvement in OS in the eribulin arm as compared with that of darcarbazine in the overall population. As per preplanned subgroup analysis, the benefit was mainly limited to the liposarcoma subgroup as compared with the LMS subgroup. Based upon this, eribulin was approved in for liposarcoma but not LMS [19].

SARC 028 study was a Phase II trial in which ten patients each of pleomorphic undifferentiated sarcoma, LMS, synovial sarcoma and liposarcoma (a total of 40 patients) were given pembrolizumab every 21 days [20]. There was no response in the LMS subgroup indicating that it might not be sensitive to immunotherapy. Similarly, in another Phase II trial evaluating single agent nivolumab, of 12 patients there was not even a single positive response [21]. Recently it has been shown that a small fraction of LMS patients might show *ALK* gene rearrangement and might be sensitive to ALK inhibitors [22]. Though the advancements in the last few years have had mixed or only marginal success, with each and every trial, the understanding of the disease is definitely getting better.

## Conclusion & future perspective

In the present patient with gastric LMS, trabectedin demonstrated an excellent disease control despite being exposed to multiple lines of chemotherapy. The well-maintained quality of life and minimal toxicity profile is worth highlighting. However, we must keep looking for biomarkers in order to optimize treatment with these novel agents.

**Executive summary**

- Metastatic leiomyosarcoma (LMS) remains a disease with a dismal outlook, with an urgent need for new therapies. The last decade has seen major advancement in terms of collaborative studies with newer agents.
- Trabectedin is a novel therapeutic agent with a pleotropic mechanism of action approved after two lines of therapy in liposarcoma and LMS.
- Trabectadin is a relatively well tolerated chemotherapeutic agent with a noncumulative toxicity profile. Major toxicities are nausea, fatigue, neutropenia, thrombocytopenia, increased alanine amino transferase, vomiting and anemia.
- In our patient with metastatic gastric LMS, the disease was nonprogressive till last follow up after receiving 17 cycles of trabectedin with minimal toxicity.
- As this patient responded to combination of gemcitabine–docetaxel, pazopanib and trabectedin it is possible that the behaviour of gastric LMS is similar to extremity LMS.
- Future research looking for biomarkers to delineate the correct subtype of patient for each therapy holds the key for better patient selection.
